# Expression and Prognostic Significance of Ferroptosis-related Proteins SLC7A11 and GPX4 in Renal Cell Carcinoma

**DOI:** 10.2174/0109298665255704230920063254

**Published:** 2023-12-06

**Authors:** Zongtao Ren, Xiaoyu Zhang, Jingya Han

**Affiliations:** 1 Department of Urology, The Fourth Hospital of Hebei Medical University, Shijiazhuang, 050011, China;; 2 Department of Nuclear Medicine, The Fourth Hospital of Hebei Medical University, Shijiazhuang, 050011, China

**Keywords:** Renal cell carcinoma, ferroptosis, SLC7A11, GPX4, prognosis, distant metastasis

## Abstract

**Background:**

The ferroptosis inhibitory gene solute carrier family 7 member 11 (SLC7A11) and glutathione peroxidase 4 (GPX4) inhibit ferroptosis in carcinoma cells. However, whether SLC7A11 and GPX4 serve as an oncogene in renal cell carcinoma (RCC) remains unclear.

**Methods:**

Immunohistochemistry (IHC) assays were performed to assess the expression of SLC7A11 and GPX4 in human RCC tissues. Clinical-pathological analysis was performed to explore the correlation between SLC7A11 and GPX4 expression. Kaplan-Meier survival analysis was performed to characterise the associations between protein expression and patient progression-free survival (PFS).

**Results:**

The upregulation of SLC7A11 and GPX4 was detected by IHC in RCC tissues compared with that in normal renal tissues. Meanwhile, the expression level of SLC7A11 and GPX4 was correlated with tumour diameter and distant metastasis (*P*<0.05). Kaplan-Meier survival analysis indicated that patients with high SLC7A11 and GPX4 expression levels exhibited worse PFS than those with low SLC7A11 and GPX4 expression levels (*P*<0.05).

**Conclusion:**

The upregulation of SLC7A11 and GPX4 expression was associated with poor prognosis in patients with RCC. SLC7A11 and GPX4 may serve as diagnostic and prognostic biomarkers for patients with RCC.

## 1 INTRODUCTION

Renal cell carcinoma (RCC) is the most common type of kidney cancer that accounts for 2%–3% of adult malignancies worldwide [1]. Clear cell RCC (CCRCC) is the most prevalent pathological type of renal cancer, and patients with CCRCC have a poorer prognosis than those with non-clear cell subtypes of RCC [2, 3]. Although surgical procedures show promising therapeutic efficacy, one-third of RCC cases experience distant metastasis or local relapse after the therapy [1]. In recent years, the prognosis of patients with advanced RCC has been improved to a certain extent with the application of molecularly targeted drugs and immune checkpoint inhibitors targeting PD-1 or its ligand PD-L1 [4, 5]. However, patients with advanced RCC become resistant to molecularly targeted drugs over the course of treatment [6]. Therefore, new potential therapeutic target molecules and prognostic markers in advanced RCC should be searched.

Ferroptosis is a newly described form of cell death resulting from the release of reactive oxygen species (ROS) to toxic levels, which involves an oxidative, iron-dependent process [7]. Glutathione peroxidase 4 (GPX4) is a key regulator of the ferroptosis process [8, 9]. The inhibition or loss of GPX4 directly leads to ferroptosis activation as a result of the accumulation of lipid peroxides. Overexpression of GPX4 confers resistance to ROS-induced cell death in tumor cells [10]. Ferroptosis is also characterised by the glutamate-cystine exchanger System Xc^−^, which plays a key role in the transport of amino acids [11]. There are at least two ways to control system Xc^−^ activity in ferroptosis. First, Solute carrier family 7 member 11 (SLC7A11) is the light chain of the System Xc^−^, which can induce ferroptosis by interacting with BECN1 (beclin 1), and the down-regulation of SLC7A11 indirectly suppresses GPX4 activity and then induces ferroptosis; Second, SLC7A11 could be regulated at the transcriptional level, and a decrease in SLC7A11 could consequently induce ferroptosis [12].

The clinical efficacy of existing cancer therapies is always unsatisfactory because of drug insensitivity or acquired resistance. In conventional treatments, inducing apoptosis or autophagy is considered a major treatment for cancer. Inducing ferroptosis can effectively kill carcinoma cells, indicating that ferroptosis can be used for the treatment of carcinoma. Ferroptosis-related proteins SLC7A11 and GPX4 participate in the regulation of the growth and proliferation of some types of carcinoma cells, such as lymphocytoma, ductal cell cancer of the pancreas, and hepatocellular carcinoma (HCC) [13-15]. Therefore, it suggests that SLC7A11 and GPX4 may serve as novel therapeutic targets for cancer therapy. However, the biological functions of ferroptosis-associated proteins SLC7A11 and GPX4 in RCC remain unclear. Therefore, the present study aims to comprehensively reveal the roles of SLC7A11 and GPX4 in RCC from the protein expression level and their potential for diagnostic and prognostic value of RCC. These findings will provide new insights into the treatment, clinical evaluation, and molecular mechanisms of RCC.

## 2 METHODS

### 2.1 Patients and Specimens

A total of 125 fresh frozen CCRCC tissues paired with corresponding normal renal tissues were obtained from patients who underwent renal tumour resection surgery in the Department of Urology, the Fourth Hospital of Hebei Medical University, between 2012 and 2016. The clinical data of these patients are relatively detailed. All patients had no history of other malignant tumours and had not received any radiotherapy or chemotherapy before surgery. All histological specimens were diagnosed as CCRCC by senior pathologists. Tissue samples were frozen in liquid nitrogen immediately after the operation. One part of these tissue samples was fixed with formalin, and the remaining part was frozen at −80 °C. The TNM tumour stages were assigned according to a modified American Joint Committee on Cancer and Union for International Cancer Control standard (version 7). Tumour grades were determined according to WHO criteria. The clinicopathological and clinical data in the study cohort are summarised in Table **1**. Of the 21 patients with distant metastases, 16 received targeted drug therapy. The study was approved by the ethics committee of the Fourth Hospital of Hebei Medical University. The participants provided their written informed consents to participate in this study.

### 2.2 Histopathology and Immunohistochemistry

Immunohistochemistry (IHC) assays were subsequently performed to detect the expression level of SLC7A11 and GPX4 protein in CCRCC and normal renal tissues fixed with formalin. Rabbit anti-human polyclonal antibodies for SLC7A11 (1:200 dilution; Jinqiao, Beijing) and GPX4 (1:200 dilution; Jinqiao, Beijing) were used to detect the expression of SLC7A11 and GPX4. The experiment was carried out following the kit instructions. Tissue sections (4μm thick) were deparaffinized with xylene, rehydrated through an ethanol series, and then immersed in 3% formaldehyde hydrogen peroxide liquid to block endogenous peroxidase. The sections were incubated with primary antibody at 4°C overnight and treated with biotin-labeled secondary antibody at 37°C for 20 min, followed by the addition of streptavidin peroxidase-conjugated antibody at 37°C for 20 min. The sections were counterstained with hematoxylin, dehydrated, transparentized and then sealed with neutral gum. All slides were examined concurrently by three experienced pathologists, who were blinded to the clinical data. The results were determined according to the staining intensity of cells and the number of positive cells.

SLC7A11 and GPX4 are mainly located in the cytoplasm of CCRCC cells. The percentage of positively stained cells was scored as 0 for <10% positive-stained cells, 1 for 10%–50% positive tumour cells and 2 for >50% positive-stained cells. The intensity staining was evaluated as 0 (no staining), 1 (moderate staining) and 2 (strong staining). The results were determined according to the staining intensity of cells and the number of positive cells. Staining scores 0, 1, and 2 were considered negative expression, while staining scores 3 and 4 were evaluated as positive expression. The diagnostic accuracy was calculated as the following: accuracy = (true positive cases + true negative cases)/total cases.

### 2.3 Follow-up

All patients were followed up in the outpatient department for 3−60 months after surgery. B-ultrasound or computed tomography (CT) was performed every 3 months after surgery for 2 years and every 6 months thereafter at the clinic or by telephone. The primary endpoint was cancer-specific mortality. The combined endpoint event was tumour-specific adverse events, including post-operative metastasis recurrence and death in patients. Progression-free survival (PFS) was defined as the period from the date of surgery to the date of disease progression or death caused by any cause. If disease progression or death had not occurred at the time of the last follow-up, PFS was considered to have been censored. The median follow-up time was 54 months. During follow-up, 9 (7.2%) patients died of other diseases. The rest of the 116 patients completed the entire follow-up process.

### 2.4 Statistical Analysis

Statistical analysis was performed using SPSS21.0 (SPSS Company, USA) and graphed using GraphPad Prism 5.0. Student’s t-test was used to compare the expression levels between different groups. The chi-square test method was used for correlation analysis of protein levels with clinic characteristics of RCC. Kaplan-Meier curves were used for survival analysis. Additionally, *P*<0.05 was considered to be significantly different.

## 3 RESULTS

### 3.1 Expression of SLC7A11 and GPX4 in RCC and Normal Renal Tissues

**Figure 1:**
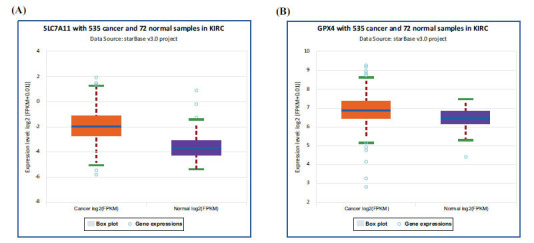
Expression level of SLC7A11 and GPX4 in the StarBase-V3.0 database. (**A**) High expression level of SLC7A11 in KIRC (*P*<0.01, KIRC also named as clear cell renal cell carcinoma). (**B**) High expression level of GPX4 in KIRC (*P*<0.01).

**Figure 2:**
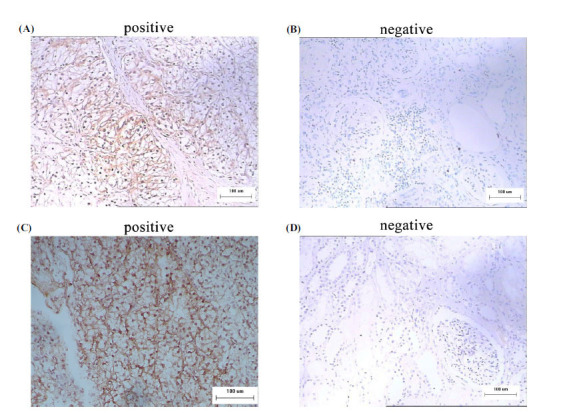
Immunohistochemical images of expression of SLC7A11 and GPX4 in RCC tissue samples and normal renal tissue samples. (**A**) Positive membrane expression of SLC7A11 in RCC (magnification, ×200). (**B**) Negative expression of SLC7A11 in normal renal tissues (magnification, ×200). (**C**) Positive membrane expression of GPX4 in RCC (magnification, ×200). (**D**) Negative expression of GPX4 in normal renal tissues (magnification, ×200).

First, the role of SLC7A11 and GPX4 in the occurrence of RCC was verified using the StarBase-V3.0 database to demonstrate that the expression level of SLC7A11 and GPX4 was upregulated in KIRC (KIRC, also named as CCRCC) tissues compared with the corresponding normal renal tissues (Figures **1A**, **1B**). Results indicated that these two proteins were upregulated in tumour tissues. Then, the protein levels of SLC7A11 and GPX4 in 125 paired RCC tissues were detected by IHC. IHC images showed that the positive staining patterns for SLC7A11 and GPX4 were observed in the cell membrane of tumour tissues (Figures **2A**, **2C**). The normal renal tissue had negative staining for SLC7A11 and GPX4 in the cell membrane (Figures **2B**, **2D**). In these patients, the positive expression rates of SLC7A11 and GPX4 in RCC tissues were 62.4% (78/125) and 57.6% (72/125), respectively and the positive rates in normal renal tissues were 29.6% (37/125) and 26.4% (33/125), respectively. The expression of these two proteins in RCC tissue and normal kidney tissue showed a significantly statistical difference (*P*<0.05). In these RCC tissues, 61 cases were SLC7A11- and GPX4-positive and 36 cases were negative, indicating that the expression of SLC7A11 was positively correlated with GPX4.

These unique IHC staining patterns illustrate that SLC7A11 and GPX4 can be used to predict clinical outcomes and can distinguish cancerous tissue from normal tissue.

### 3.2 Correlation Analysis between SLC7A11, GPX4 Expression Level, and Clinicopathological Factors of Patients with RCC

We analysed the relationship between SLC7A11, GPX4 and clinicopathologic features. Notably, the high expression levels of SLC7A11 and GPX4 were related to tumour diameter, distant metastasis, and clinical stage of RCC (*P*<0.05) but not to age, sex, lymph node metastasis, or pathological differentiation (*P*>0.05, Table **1**).

### 3.3 Kaplan-Meier Survival Analysis between SLC7A11, GPX4 Expression Level, and Patient Survival

**Figure 3:**
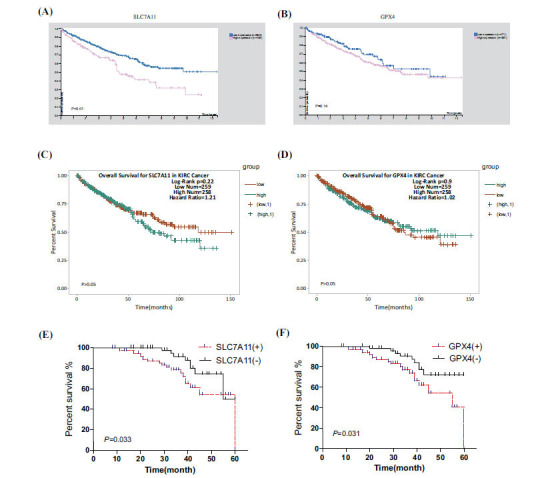
Prognostic value of SLC7A11 and GPX4 expression levels. (**A, B**) Survival difference between high and low SLC7A11, GPX4 expressions in the Human Protein Atlas. (**C, D**) Survival difference between high and low SLC7A11 and GPX4 expression levels in the StarBase-V3.0 database. (**E, F**) Kaplan-Meier survival analysis of patients with renal cell carcinoma based on SLC7A11 and GPX4 expression.

Furthermore, we tested the prognostic value of SLC7A11 and GPX4 in patients with RCC in The Human Protein Atlas database. As shown in the human protein atlas database, the high expression of SLC7A11 resulted in poor prognosis (*P*<0.01, Figure **3A**), but GPX4 did not affect the prognosis of RCC (*P*=0.14, Figure **3B**). Therefore, SLC7A11 was identified as a prognostic factor. However, a different result was obtained in the StarBase-V3.0 database, and SLC7A11 and GPX4 were not identified as prognostic factors (*P*>0.05, Figures **3C**, **3D**).

In the Kaplan-Meier survival analysis, patients with positive SLC7A11 expression had significantly lower PFS than patients with negative SLC7A11 expression (95%CI: 46.712~53.119, *P*<0.05, Figure **3E**); in addition, compared with patients with RCC having negative GPX4, patients with positive GPX4 had decreased PFS (95%CI: 46.712~53.119, *P*<0.05, Figure **3F**). Therefore, high SLC7A11 and GPX4 expression led to a poor prognostic effect for RCC patients.

## 4 DISCUSSION

RCC represents approximately 90% of all malignancies of the kidney, while clear cell CCRCC accounts for 70%–80% of all RCC cases and also is one of the most aggressive subtypes [16]. Although RCC can be completely removed by surgery, considering the resistance to chemotherapy and radiotherapy, patients with RCC are prone to local recurrence or distant metastasis [17]. Although anti-angiogenic agents, receptor-targeted therapy and immune checkpoint inhibition are effective for the treatment of advanced RCC, the five-year survival rate for patients with distant metastases is only 10% [18, 19]. Therefore, new treatment targets and effective treatment methods should be developed.

Ferroptosis is a form of programmed cell death identified in 2012, which involves the production of iron-dependent ROS and is distinct from apoptosis, necroptosis and autophagy in both morphological changes and biochemical processes [20, 21]. Ferroptosis plays an important role in the occurrence of some kinds of tumours, including HCC [15]. Although the detailed molecular regulatory mechanisms of ferroptosis are incompletely understood, some molecules, such as SLC7A11 and glutathione peroxidase 4 (GPX4), regulate ferroptosis by affecting iron metabolism and lipid peroxidation [21]. The system x_c_^−^ cystine-glutamate anti-porter and GPX4 are two of the validated targets for inducing ferroptosis. SLC7A11 is a key component of a plasma membrane antiporter (the x_c_^−^ system) that mediates Na^+^-independent cellular uptake of extracellular cystine in exchange for intracellular glutamate [22]. SLC7A11 overexpression promotes cancer progression by suppressing ferroptosis. SLC7A11 overexpression is observed in many human cancers. BRCA1-associated protein 1 (BAP1) could promote ferroptosis by blocking the expression of SLC7A11. GPX4 is a special enzyme that regulates ferroptosis by targeting the antioxidant system, and glutathione (GSH) is an essential cofactor in its activation. By depleting the intracellular GSH pool, the ferroptosis inducers erastin reduce GPX4 activity and elevate ROS levels, ultimately leading to cell ferroptosis. [23]. The inhibition or loss of GPX4 directly leads to ferroptosis activation as a result of the accumulation of lipid peroxides [10]. Liu *et al.* found that the downregulation of SLC7A11 could indirectly cause the suppression of GPX4 activity and then lead to ferroptosis [24]. The upregulation of SLC7A11 increases the expression of GPX4 and inhibits the activation of ferroptosis.

To investigate whether ferroptosis plays a role in renal cell carcinoma, we carried out this experiment to preliminarily investigate the expression of ferroptosis-associated proteins in RCC. In the study, we detected the expression of SLC7A11 and GPX4 proteins in 125 cases of RCC tissues and corresponding normal renal tissues by IHC. As shown in the images, the RCC specimens have greater cell density than the normal renal specimens. IHC staining indicates that positive staining patterns for SLC7A11 and GPX4 were observed in the cytoplasm of RCC tissues. SLC7A11 protein was positively expressed in 78 tumour tissues, but only 37 in normal tissues; meanwhile, GPX4 protein was positively expressed in 72 tumour tissues, while only 33 cases exhibited positive staining in normal tissues. These findings agree with a previous report, in which SLC7A11 was significantly upregulated in both clinical specimens and cell lines of breast cancer [25]. Also, Yang *et al.* analysed the expression of GPX4 in 50 pairs of colon tumour tissues by performing IHC. They found that GPX4 was positively expressed in the colon tumour tissues [26]. Based on the positive expression of SLC7A11 and GPX4 in RCC tissues, we preliminarily concluded that ferroptosis may play a role in the occurrence and progression of renal cell carcinoma.

Furthermore, we analysed the correlation between SLC7A11 and GPX4. In these RCC tissues, 61 cases were SLC7A11- and GPX4-positive and 36 cases were negative. We found a positive correlation between the expression of the two proteins. Therefore, SLC7A11, as an upstream protein, may regulate the expression of GPX4 to a certain extent. In addition, follow-up studies will be conducted to further clarify how SLC7A11 affects the expression of GPX4. Lee *et al.* found that GPX4 was highly expressed in breast cancer tissues compared with matched normal samples, which was correlated with the increased expression of the xCT subunits SLC7A11; Erastin, an inducer of ferroptosis, depleted levels of the antioxidant selenoproteins GPX4 in breast cancer cells by inhibiting xCT-dependent extracellular reduction, which further demonstrated the synergistic role of SLC7A11/GPX4 in regulating ferroptosis [27]. To verify the expression level and prognostic roles of SLC7A11 and GPX4 in pan-cancer, Shi *et al.* [28] analyzed these two genes by using GEPIA and Kaplan–Meier databases. They found that SLC7A11 and GPX4 are dysregulated in many types of cancers and may serve as candidate prognostic biomarkers, such as colorectal cancer and lung cancer. To further clarify the biofunctions of SLC7A11 and GPX4 in renal carcinoma cells, we will perform a series of experiments *in vitro* in the near future.

SLC7A11 has oncogenic functions in carcinoma. For example, Polewski *et al.* found that SLC7A11 was overexpressed in glioblastoma multiforme and contributed to tumorigenesis, tumor progression, and resistance to chemotherapy [29]. Meanwhile, Robert *et al.* demonstrated that patients with reduced SLC7A11 expression have longer survival than patients with elevated SLC7A11 levels and the high expression level of SLC7A11 is associated with accelerated tumor growth and predicts poor survival in patients with malignant glioma [30]. In addition, Shen *et al.* identified that patients with high SLC7A11 expression levels in papillary thyroid carcinoma exhibited poorer survival than those with low SLC7A11 expression levels [31]. Besides, Zhang *et al.* found that GPX4 was negatively associated with the prognosis of patients with cholangiocarcinoma and lung squamous cell carcinoma [32]. These results indicate that SLC7A11 and GPX4 are potentially useful prognostic biomarkers. In the present study, we first analysed the relationship between SLC7A11, GPX4 and clinicopathologic features of patients with RCC. Notably, the expression levels of SLC7A11 and GPX4 were related to tumour diameter and distant metastasis but not to age, sex, lymph node metastasis, or pathological differentiation.

Moreover, SLC7A11 expression was positively correlated with GPX4 in RCC tissues. Furthermore, we grouped the patients into positive and negative groups and then analysed whether the expression of SLC7A11 and GPX4 was associated with the prognosis of RCC patients. The K-M curve indicates that SLC7A11 and GPX4 were negatively associated with the PFS of patients with RCC. The results of our study are consistent with those of a previous study: SLC7A11 was significantly upregulated in RCC, and overexpression of SLC7A11 conferred a worse prognosis and was identified as an independent prognostic factor [33]. However, our results differ from those in the Human Protein Atlas and StarBase-V3.0 database, in which GPX4 was not a prognostic factor in RCC. The reason for this difference is that a few cases that we studied had a short follow-up time on patients. These findings suggest that SLC7A11 and GPX4 might have great prognostic values in RCC patients.

Although the correlation between SLC7A11 and GPX4 has been demonstrated through the current study, several limitations are still observed. First, this study was not conducted *in vitro*. The specific regulatory mechanisms by which SLC7A11 regulates GPX4 still need to be verified. We will continue to explore the changes in ROS caused by changing the expression level of SLC7A11 or GPX4 *in vitro* to further clarify the relationship of SLC7A11 or GPX4 with ferroptosis. Second, the RCC sample was relatively small, and clinical data of the samples were not complete, such as, the effect of treatment on survival of patients with advanced renal carcinoma was not considered. Additionally, it is imperative for future investigations to incorporate prospective data from renal cell carcinoma (RCC) patients and conduct *in vitro* experiments to validate the impact of SLC7A11 or GPX4 on the malignant biological behavior of RCC cells, thereby reinforcing the findings presented in this study.

## CONCLUSION

Collectively, the findings of this study illustrate that SLC7A11 and GPX4 were upregulated in RCC. The high expression of SLC7A11 and GPX4 was associated with poor prognosis in RCC patients. Therefore, SLC7A11 and GPX4 may serve as potential therapeutic targets for RCC patients.

## Figures and Tables

**Table 1:** The relationship between expression of SLC7A11、GPX4 and Clinical characteristics of RCC.

**Group**	**N**	**SLC7A11**				**GPX4**			
		**+**	**-**	**Positive rate**	***P***	**+**	**-**	**Positive rate**	***P***
RCC	125	78	47	62.4%		72	53	57.6%	
**Gender**									
Male	76	45	31	59.2%	0.359	40	36	52.6%	0.162
Female	49	33	16	67.3%		32	17	65.3%	
**Age**									
≤60	67	42	25	62.7%	0.943	37	30	55.2%	0.563
>60	58	36	22	62.1%		35	23	60.3%	
**Pathologic Grade**									
Well/mode	89	58	31	65.2%	0.315	49	40	55.1%	0.366
Poorly	36	20	16	55.6%		23	13	63.9%	
**Tumor Diameter**									
≤7.0cm	77	39	38	50.6%	0.001	39	38	50.6%	0.046
>7.0cm	48	39	9	81.2%		33	15	68.8%	
**Lymph Node**									
yes	15	7	8	46.7%	0.18	8	7	53.3%	0.841
no	110	71	39	64.5%		64	46	58.2%	
**Distant Metastasis**									
yes	21	18	3	85.7%	0.016	17	4	81.0%	0.018
no	104	60	44	57.7%		55	49	52.9%	
**Clinical Stage**									
I+II	82	46	36	56.1%	0.045	41	41	50.0%	0.018
III+IV	43	32	11	74.4%		31	12	72.1%	

## Data Availability

The data and supportive information are available within the article.

## LIST OF ABBREVIATIONS

CCRCC: Clear Cell Renal Cell Carcinoma
CT: Computed Tomography
GPX4: Glutathione Peroxidase 4
HCC: Hepatocellular Carcinoma
IHC: Immunohistochemistry
PD-1: Programmed Cell Death-1
ROS: Reactive Oxygen Species
PFS: Progression-free Survival
RCC: Renal Cell Carcinoma
SLC7A11: Solute Carrier Family 7 Member 11
