# Supplementary material for: Expression and Prognostic Significance of Ferroptosis-related Proteins SLC7A11 and GPX4 in Renal Cell Carcinoma
Source: Protein Pept Lett. 2023 Dec 6;30(10):868–76. doi: 10.2174/0109298665255704230920063254 (PMC10788919; doi:10.2174/0109298665255704230920063254)
Supplement: Supplementary file 1 — Supplementary material is available on the publisher’s website along with the published article. [file PPL-30-868_SD1.pdf]

Supplementary Materials

Expression and Prognostic Significance of Ferroptosis-related Proteins SLC7A11 and GPX4 in Renal Cell Carcinoma

Zongtao Ren[1], Xiaoyu Zhang[1] and Jingya Han[2,*]

[1]Department of Urology, The Fourth Hospital of Hebei Medical University, Shijiazhuang, 050011, China; [2]Department of Nuclear Medicine, The Fourth Hospital of Hebei Medical University, Shijiazhuang, 050011, China

TABLE S1

	Item No.	Recommendation	Page No.	Relevant text from manuscript
**Title and abstract**	1	(*a*) Indicate the study's design with a commonly used term in the title or the abstract	Page 2	Kaplan-Meier survival analyse was performed to characterise progression-free survival (PFS)
		(*b*) Provide in the abstract an informative and balanced summary of what was done and what was found	Page 2	The upregulation of SLC7A11 and GPX4 was detected by IHC in RCC tissues.<br>The upregulation of SLC7A11 and GPX4 expression was associated with poor prognosis in patients with RCC.
Background/rationale	2	Explain the scientific background and rationale for the investigation being reported	Page 2-3	The clinical efficacy of existing cancer therapies is always unsatisfactory because of drug insensitivity or acquired resistance.<br>Inducing ferroptosis can effectively kill carcinoma cells, indicating that ferroptosis can be used for the treatment of carcinoma. Ferroptosis participates in the regulation of the growth and proliferation of some types of carcinoma cells. However, the biological functions of ferroptosis associated proteins SLC7A11 and GPX4 in RCC remain unclear.
Objectives	3	State specific objectives, including any prespecified hypotheses	Page 3	the present study aims to study the expression level of SLC7A11 and GPX4 and their potential for diagnosis and prognosis of RCC.
Study design	4	Present key elements of study design early in the paper	Page 4	The combined endpoint event was tumour-specific adverse events. If disease progression or death had not occurred at

 © 2023 Bentham Science Publishers

				the time of the last follow-up, PFS was considered to have been censored.
Setting	5	Describe the setting, locations, and relevant dates, including periods of recruitment, exposure, follow-up, and data collection	Page 4	All patients were followed up in the outpatient department for 3−60 months after surgery.<br>The primary end point was cancer-specific mortality.<br>B-ultrasound or computed tomography (CT) was performed every 3 months after surgery for 2 years and every 6 months thereafter at the clinic or by telephone.
Participants	6	a) ***Cohort study***—Give the eligibility criteria, and the sources and methods of selection of participants. Describe methods of follow-up<br>b) ***Case-control study***—Give the eligibility criteria, and the sources and methods of case ascertainment and control selection. Give the rationale for the choice of cases and controls<br>c) ***Cross-sectional study***—Give the eligibility criteria, and the sources and methods of selection of participants	Page 3	All patients had no history of other malignant tumours and had not received any radiotherapy or chemotherapy before surgery. All histological specimens were diagnosed as CCRCC by senior pathologists.
		d) ***Cohort study***—For matched studies, give matching criteria and number of exposed and unexposed<br>*e)* ***Case-control study***—For matched studies, give matching criteria and the number of controls per case	N/A	N/A
Variables	7	Clearly define all outcomes, exposures, predictors, potential confounders, and effect modifiers. Give diagnostic criteria, if applicable	Page 3, 4	Add up the two points above. Staining scored 0, 1 and 2 was considered negative-expression, while staining scored 3 and 4 was evaluated as positive expression. Progression-free survival (PFS) was defined as the period from the date of surgery to the date of disease progression or death caused by any cause
Data sources/ measurement	8*	For each variable of interest, give sources of data and details of methods of assessment (measurement). Describe comparability of assessment methods if there is more than one group	Page 4	If disease progression or death had not occurred at the time of the last follow-up, PFS was considered to have been censored.
Bias	9	Describe any efforts to address potential sources of bias	N/A	N/A
Study size	10	Explain how the study size was arrived at	Page 3	The clinical data of these patients are relatively detailed.

Quantitative variables	11	Explain how quantitative variables were handled in the analyses. If applicable, describe which groupings were chosen and why	Page 3	The results were determined according to the staining intensity of cells and the number of positive cells.
Statistical methods	12	(*a*) Describe all statistical methods, including those used to control for confounding	Page 4	The chi-square test method was used for correlation analysis of protein levels with

				clinic characteristics of RCC. Kaplan-Meier curves were used for survival analysis.
		(*b*) Describe any methods used to examine subgroups and interactions	Page 4	The chi-square test method was used for correlation analysis of protein levels with clinic characteristics of RCC.
		(*c*) Explain how missing data were addressed	N/A	N/A
		(*d*) *Cohort study*—If applicable, explain how loss to follow-up was addressed<br>*Case-control study*—If applicable, explain how matching of cases and controls was addressed<br>*Cross-sectional study*—If applicable, describe analytical methods taking account of sampling strategy	N/A	N/A
		(*e*) Describe any sensitivity analyses	N/A	N/A
Participants	13*	(a) Report numbers of individuals at each stage of study—eg numbers potentially eligible, examined for eligibility, confirmed eligible, included in the study, completing follow-up, and analysed	Page 4	Duing follow up, 9 (7.2%) patients died of other diseases. The rest of 116 patients completed the entire follow-up process.
		(b) Give reasons for non-participation at each stage	Page 4	Duing follow up, 9 (7.2%) patients died of other diseases.
		(c) Consider use of a flow diagram		
Descriptive data	14*	(a) Give characteristics of study participants (eg demographic, clinical, social) and information on exposures and potential confounders	Page 4	We analysed the relationship between SLC7A11, GPX4 and clinicopathologic features. Table 1
		(b) Indicate number of participants with missing data for each variable of interest	N/A	N/A
		(c) *Cohort study*—Summarise follow-up time (eg, average and total amount)	Page 4	The median follow-up time was 54 months.
Outcome data	15*	*Cohort study*—Report numbers of outcome events or summary measures over time	N/A	N/A
		Case-control study—Report numbers in each exposure category, or summary measures of exposure	Page 4	In these patients, the positive expression rates of SLC7A11 and GPX4 in RCC tissues were 62.4% (78/125) and 57.6% (72/125), respectively and the positive rates in normal renal tissues were 29.6% (37/125) and 26.4% (33/125), respectively
		Cross-sectional study—Report numbers of outcome events or summary measures	N/A	N/A
Main results	16	(*a*) Give unadjusted estimates and, if applicable, confounder-adjusted estimates and their precision (eg, 95% confidence interval). Make clear which confounders were adjusted for and why they were included	Page 5	In the Kaplan-Meier survival patients with positive SLC7A11 expression had significantly lower PFS than patients with negative SLC7A11 expression (95%CI: 46.712~53.119, $P<0.05$, Fig. 3e); in addition, compared with patients with RCC having negative GPX4, patients with positive GPX4 had decreased PFS (95%CI: 46.712~53.119, $P<0.05$, Fig. 3f).

		(*b*) Report category boundaries when continuous variables were categorized	N/A	N/A
		(*c*) If relevant, consider translating estimates of relative risk into absolute risk for a meaningful time period	N/A	N/A
Other analyses	17	Report other analyses done—eg analyses of subgroups and interactions, and sensitivity analyses	Page 4	In these RCC tissues, 61 cases were SLC7A11- and GPX4-positive and 36 cases were negative, indicating that the expression of SLC7A11 was positively correlated with GPX4.
Key results	18	Summarise key results with reference to study objectives	Page 7	The high expression of SLC7A11 and GPX4 was associated with poor prognosis in RCC patients.
Limitations	19	Discuss limitations of the study, taking into account sources of potential bias or imprecision. Discuss both direction and magnitude of any potential bias	Page 7	First, this study has not been conducted *in vitro*, the specific regulatory mechanisms by which SLC7A11 regulate GPX4 still needs to be verified. We will continue to explore the changes in ROS caused by changing the expression level of SLC7A11 or GPX4 in vitro to further clarify the relationship of SLC7A11 or GPX4 with ferroptosis. Second, the RCC sample is relatively small, and clinical data of the samples are not complete.
Interpretation	20	Give a cautious overall interpretation of results considering objectives, limitations, multiplicity of analyses, results from similar studies, and other relevant evidence	Page 7	Although the correlation between SLC7A11 and GPX4 has been demonstrated through the current study, several limitations are still observed. Therefore, future studies should include the prospective data of RCC patients and *in vitro* experimental verification to strengthen the findings of this study.
Generalisability	21	Discuss the generalisability (external validity) of the study results	Page 6	SLC7A11 has oncogenic functions in carcinoma. For example, the overexpression of SLC7A11 resulted in decreased migration and invasion in glioblastoma [29]. The high expression level of SLC7A11 is associated with accelerated tumour growth and predicts poor survival in patients

				with malignant glioma [30].
Funding	22	Give the source of funding and the role of the funders for the present study and, if applicable, for the original study on which the present article is based	Page 7	**Funding** No funding was received to assist with the preparation of this manuscript.
